# Application of unilateral biportal endoscopy in minimally invasive treatment of non-aggressive spinal benign tumors: technical note and preliminary clinical study

**DOI:** 10.3389/fsurg.2026.1878422

**Published:** 2026-07-02

**Authors:** Qiang Ren, Liang-Jie He, Qi-Chang Li, Jia-Nan Zhang, Shi-Chang Dai, Wen-Ge He, Liang Chen

**Affiliations:** 1 Department of Bone and Soft Tissue Oncology, Chongqing University Cancer Hospital, Chongqing, China; 2 Chongqing Key Laboratory of Translational Research for Cancer Metastasis and Individualized Treatment, Chongqing University Cancer Hospital, Chongqing, China

**Keywords:** minimally invasive treatment, non-aggressive spinal benign tumors, spinal surgery, tumor curettage, unilateral biportal endoscopy (UBE)

## Abstract

**Objective:**

To introduce the technical protocol of unilateral biportal endoscopy (UBE) in the treatment of patients with non-aggressive spinal benign tumors and evaluate its clinical efficacy and safety.

**Methods:**

A retrospective analysis was conducted on 14 patients (6 males, 8 females) with pathologically confirmed spinal benign tumors who underwent UBE surgery at Chongqing University Cancer Hospital between August 2021 and December 2023. Operative duration, total perioperative blood loss, and postoperative complications were recorded. Back pain visual analog scale (VAS) scores were compared preoperatively and at postoperative day 1, 3 months, 6 months, and the final follow-up. Preoperative and predischarge CT imaging findings were also assessed.

**Results:**

All 14 patients successfully underwent UBE surgery, with a mean operative duration of 77.5 ± 21.2 min and a mean blood loss of 17.5 ± 11.7 mL. Postoperative CT confirmed adequate lesions resection. Patients were followed up for 10–30 months (mean: 16.6 ± 5.4 months). VAS scores at all postoperative timepoints showed significant improvement compared to preoperative values (*P* < 0.05). No tumor recurrence was observed during follow-up, and all patients resumed normal daily activities.

**Conclusion:**

UBE is a safe and effective surgical option for non-aggressive spinal benign tumors, achieving satisfactory lesions resection and favorable clinical outcomes.

## 1 Introduction

Although primary benign spinal tumors are rare, their unique anatomical location often causes severe clinical consequences. Studies indicate most lesions remain asymptomatic and clinically undetected, leading to significant underdiagnosis (1). Common pathological types include hemangiomas, lipomas, osteosclerosis, aneurysmal bone cysts, osteoid osteomas, and osteoblastomas (2). Despite being histologically benign, these tumors can exhibit biologically aggressive behavior, potentially causing progressive destruction of vertebral bodies, pedicles, or posterior elements. This can ultimately lead to spinal mechanical instability or compression of the spinal cord and nerve roots.

For symptomatic patients with benign spinal tumors, surgical intervention remains the primary approach. It alleviates symptoms, prevents tumor progression, and avoids subsequent spinal instability or neurological deficits. Traditional techniques typically involve tumor removal via intralesional curettage or *en bloc* resection, combined with internal fixation to restore spinal stability (3–5). However, these procedures often cause significant trauma, substantial blood loss, and prolonged postoperative recovery. Clinically, lesions are evaluated with the Enneking classification system, and benign tumors are categorized into three subtypes. Stage S1 lesions are inactive and asymptomatic with arrested growth and intact capsule; Stage S2 lesions present mild symptoms, well-defined margins and a low recurrence risk; Stage S3 lesions grow rapidly with ruptured or absent capsules, adjacent soft tissue invasion and markedly elevated recurrence rate (6, 7). In this study, the included non-aggressive spinal benign tumors were limited to Enneking Stage S1 and S2 lesions. Considering the high recurrence risk of Stage S3 lesions, open surgical resection is recommended for such cases.

Recent advances in spinal endoscopic technology and instrumentation have increased academic interest in unilateral biportal endoscopic (UBE) technique (8). Our preliminary research first reported successful single-portal endoscopic treatment of benign spinal tumors, demonstrating promising outcomes with minimally invasive management (9). While UBE efficacy for degenerative spinal conditions is well-established, its application for benign spinal tumors remains undocumented.

Therefore, exploring more precise, minimally invasive approaches for non-aggressive benign spinal tumors is necessary. Expanding UBE technology applications represents an important advancement in minimally invasive spinal tumor management. This study aims to evaluate UBE safety and efficacy for non-aggressive spinal benign tumors. Our ultimate goal is to establish UBE as a viable, safe, and effective surgical option.

## 2 Materials and methods

### 2.1 Study subjects and data collection

This study received approval from the Ethics Committee of Chongqing University Cancer Hospital. Written informed consent was obtained from all participants for research purposes and related imaging use. Given the retrospective design, we included patients who underwent UBE surgery for pathologically confirmed benign spinal tumors at our institution between August 2021 and December 2023. We systematically reviewed electronic medical records, surgical documentation, and radiographic data. The final cohort comprised 14 eligible cases.

We recorded the following parameters: demographic characteristics (age, sex); tumor type; tumor stage according to the Enneking classification system; vertebral level of involvement; anatomical extent [classified using the Weinstein–Boriani–Biagini (WBB) system]; clinical symptoms; intraoperative metrics (surgical duration, blood loss); preoperative and postoperative visual analog scale (VAS) scores at designated intervals; and follow-up duration. All data appear in Table 1.

**Table 1:** Clinical characteristics data of all patients with spinal benign tumors.

Patient	Age/Gender	Tumor type^a^	Lesion segment	Location (WBB classification)	Enneking stage	Symptom	Operation time (min)	Estimated blood loss (mL)	Follow-up duration (months)	Complications
1	30/M	O	T12	Sectors 3–4, layer C	S2	Back pain	105	35	13	None
2	26/M	O	T10	Sectors 8–10, layer C	S2	Back pain	90	40	12	None
3	21/M	ABC	T11	Sectors 3–5, layer C	S2	Back pain	130	30	14	None
4	29/M	ABC	L2	Sectors 4–5, layer C	S2	Back pain	80	20	18	None
5	30/F	ABC	L4	Sectors 9–10, layer C	S2	Back pain	90	30	16	None
6	49/F	BFH	L2	Sectors 8–10, layer C	S2	Back pain	60	10	30	None
7	14/F	BFH	L1	Sectors 3–4, layer C	S2	Back pain	70	10	15	None
8	23/F	FD	L3	Sectors 3–4, layer C	S2	Back pain	65	10	16	None
9	42/F	FD	L5	Sectors 8–9, layer C	S2	Back pain	70	10	24	None
10	17/F	SBC	T12	Sectors 3–5, layer C	S2	Back pain	65	5	10	None
11	23/F	SBC	L1	Sectors 4–5, layer C	S2	Back pain	70	10	22	None
12	42/M	SBC	T11	Sectors 9–10, layer C	S2	Back pain	65	20	14	None
13	35/M	OO	L5	Sectors 4, layer C	S2	Back pain	45	5	17	None
14	17/F	OO	L4	Sectors 3–4, layer C	S2	Back pain	80	10	12	None

a ABC, aneurysmal bone cyst; OO, osteoid osteoms; SBC, Simple bone cyst; FD, fibrous dysplasia; BFH, benign fibrous histiocytoma; O, osteoblastoma.

### 2.2 Surgical procedure

All surgeries were performed by Dr. Liang Chen under general anesthesia. The specific surgical procedures were as follows: Since the lesions of enrolled patients were located in the pedicle and ipsilateral vertebral body (WBB zones 3–5 or 8–10), the pedicle approach was selected for the surgery. The involved pedicle was localized via fluoroscopy. Two incisions (approximately 1.5 cm in length) were made at the body surface projection of the involved pedicle and 1.5 cm cephalad to it, respectively, to establish the observation channel (cephalad) and operating channel (caudal); all operations were performed through the operating channel. A Kirschner wire was inserted into the lesion through the operating channel, and fluoroscopy confirmed that the puncture path of the Kirschner wire was in the central area of the lesion with the needle tip reaching the distal end of the lesion (Figure 2). Under endoscopy, surrounding soft tissues were cleared to expose bony structures. After identifying the anchor point of the Kirschner wire on the cortical bone, a burr was used under endoscopy to perform subperiosteal grinding along the anchor point into the pedicle until reaching the distal end of the lesion. Tumor tissues within the pedicle and involved vertebral body were visualized endoscopically. After hemostasis with a radiofrequency electrode, the tumor tissues were thoroughly removed using a grasper. Subsequently, a high-speed burr was used to perform 360° grinding on the inner wall of the pedicle and the involved vertebral body until normal bone was exposed. Chemical inactivation was then performed using absolute ethanol, hydrogen peroxide, and concentrated iodophor. After chemical inactivation, the lesion was cleared again, and the lesion cavity was ablated with a radiofrequency electrode to ensure complete clearance of the lesion. Thereafter, allogeneic bone was implanted into the bony cavity of the lesion, and one negative-pressure drainage tube was placed at the incision site (Figure 3).

### 2.3 Postoperative management

Prophylactic antibiotics were administered once postoperatively. The drainage tube was removed when the output was <20 mL. A lumbar brace was permitted on postoperative day 1, with gradual sitting and ambulatory rehabilitation under protection. Pre-discharge CT was performed to evaluate surgical outcomes. Strenuous activities were prohibited for the first 3 postoperative months.

### 2.4 Statistical analysis

Data were analyzed using SPSS 23.0. Normally distributed continuous variables were expressed as mean ± standard deviation and compared using paired *t*-tests. Non-normally distributed data were presented as median (interquartile range) and analyzed with Wilcoxon signed-rank tests. A two-tailed *P* < 0.05 was considered statistically significant.

## 3 Results

All 14 patients (6 male, 8 female; mean age 28.4 ± 10.5 years) successfully underwent surgery (Table 2). Mean operative time was 77.5 ± 21.2 min with blood loss of 17.5 ± 11.7 mL. Follow-up ranged 10–30 months (mean 16.6 ± 5.4 months). All incisions healed primarily without complications (infection, nerve injury, or others). Pathology confirmed these tumor types: benign fibrous histiocytoma, osteoblastoma, osteoid osteomas, aneurysmal bone cyst, simple bone cyst, and fibrous dysplasia. Back pain scores significantly improved postoperatively vs. preoperative at all timepoints (Table 3). No recurrence occurred during follow-up. All patients resumed normal activities.

**Table 2:** Patient characteristics.

Variables	Values
Mean age (years)	28.4 ± 10.5
Gender, *n*	
Male	6
Female	8
Mean follow—up period (months)	16.6 ± 5.4
Diagnoses, *n*	
Benign fibrous histiocytoma	2
Osteoblastoma	2
Osteoid osteoms	2
Aneurysmal bone cyst	3
Simple bone cyst	3
Fibrous dysplasia	2
Mean intraoperative blood loss (mL)	17.5 ± 11.7
Mean operation time (min)	77.5 ± 21.2
Postoperative complications	None
Recurrence	None

**Table 3:** Visual analogue scale (VAS) scores for back pain at different time points.

Time point	VAS score, mean ± SD	*P*-value (vs. preoperative)
Preoperative	4.6 ± 0.8	–
Postoperative 1 day	3.1 ± 0.8	<0.05
Postoperative 3 months	1.4 ± 0.6	<0.01
Postoperative 6 months	0.6 ± 0.5	<0.01
Last follow-up	0.8 ± 0.4	<0.01

### 3.1 Typical case presentation

**Figure 1:**
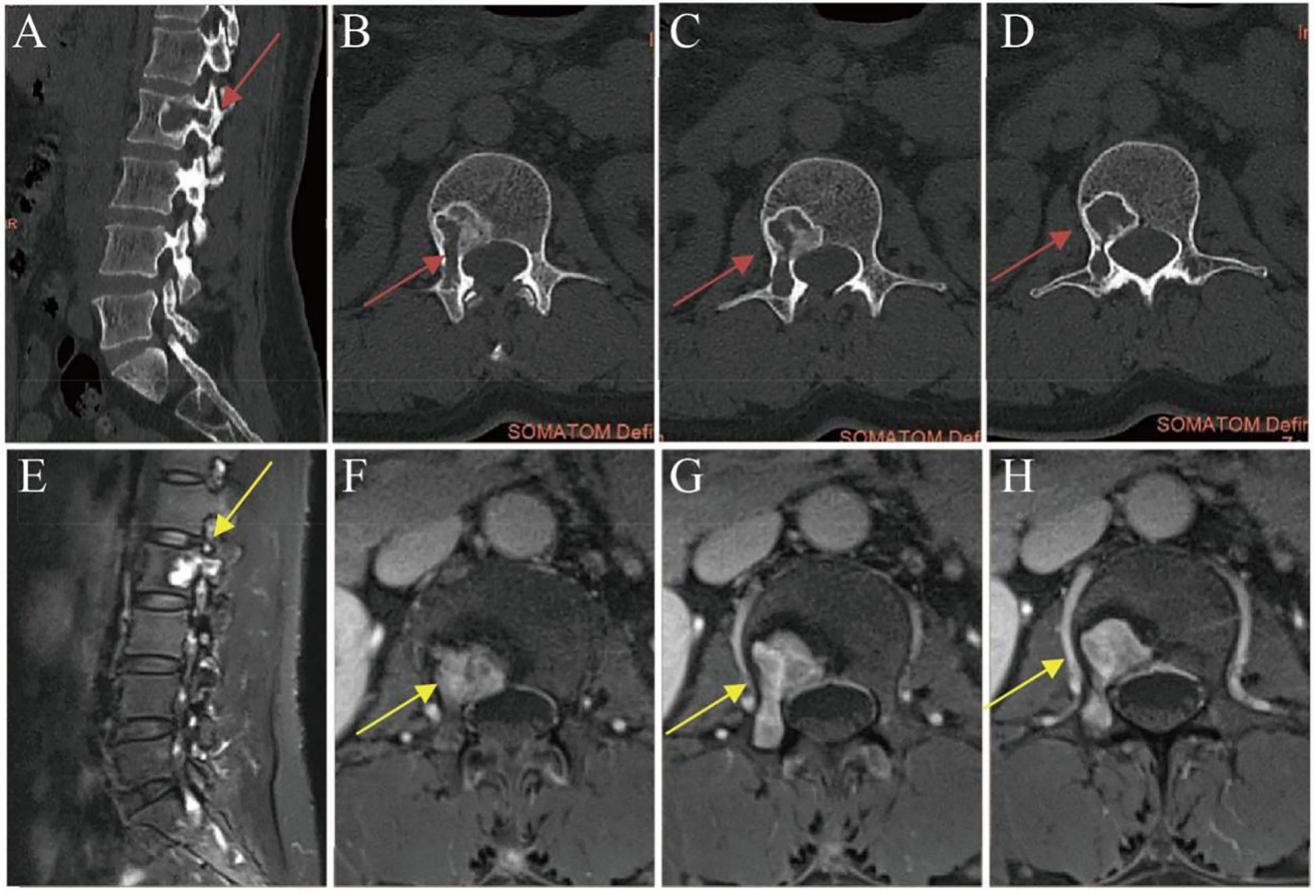
**(A–D)** Represent preoperative CT scans: irregular osseous destruction area is observed in the L2 vertebral body and its right appendage (red arrows). Images **(E–H)** show preoperative MRI scans: Irregular abnormal signal changes are visible in the L2 vertebral body and its right appendage, with T2 fat-suppressed images demonstrating uneven high signal intensity (yellow arrows).

A 49-year-old woman presented with a 1-year history of recurrent low back pain. Admission VAS score for low back pain was 5/10, without radiculopathy or neurological deficits. Physical examination showed lumbar percussion tenderness. CT (Figures 1A–D) revealed a 3.0 cm × 1.9 cm irregular osteolytic lesion with sclerotic margins in the L2 vertebral body and right pedicle. No periosteal reaction or soft-tissue swelling was noted. MRI (Figures 1E–H) showed a well-defined lesion: isointense on T1WI, heterogeneously hyperintense on T2-weighted fat-saturated sequences. A hypointense rim surrounded the lesion on all sequences. Post-contrast imaging demonstrated marked heterogeneous enhancement. Serum tumor markers were negative. Preoperative needle biopsy confirmed benign histology without malignancy.

**Figure 2:**
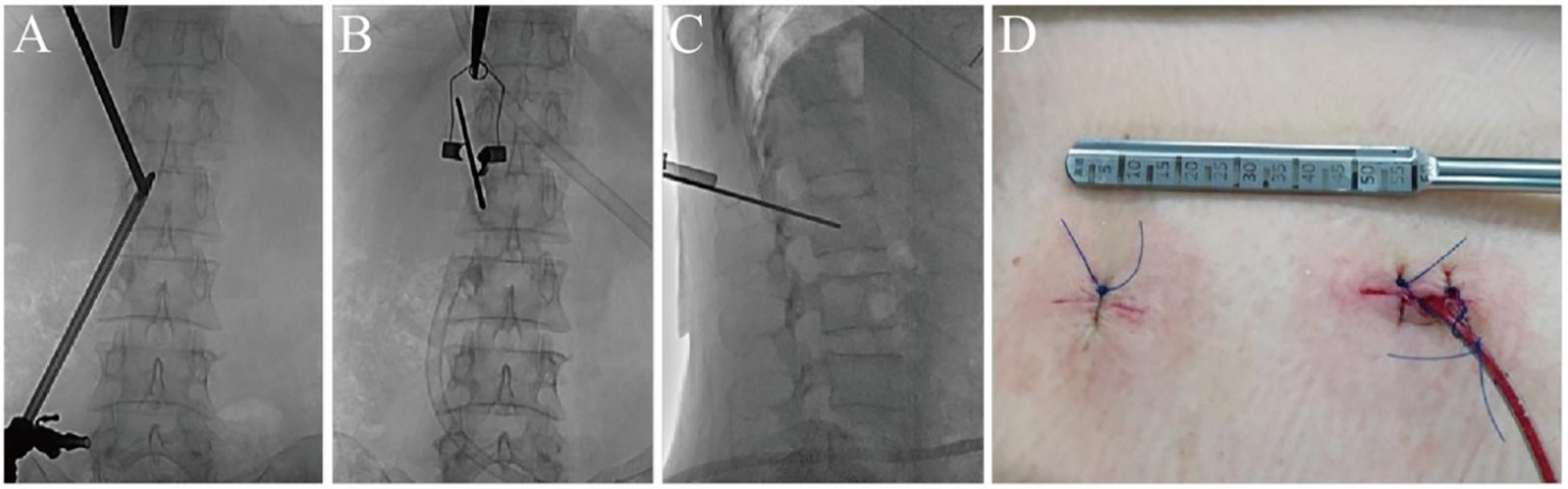
**(A)** Intraoperative fluoroscopy placement of UBE endoscope. **(B,C)** Fluoroscopy to confirm the surgical operating area as the lesion area. **(D)** Incision after postoperative closure.

**Figure 3:**
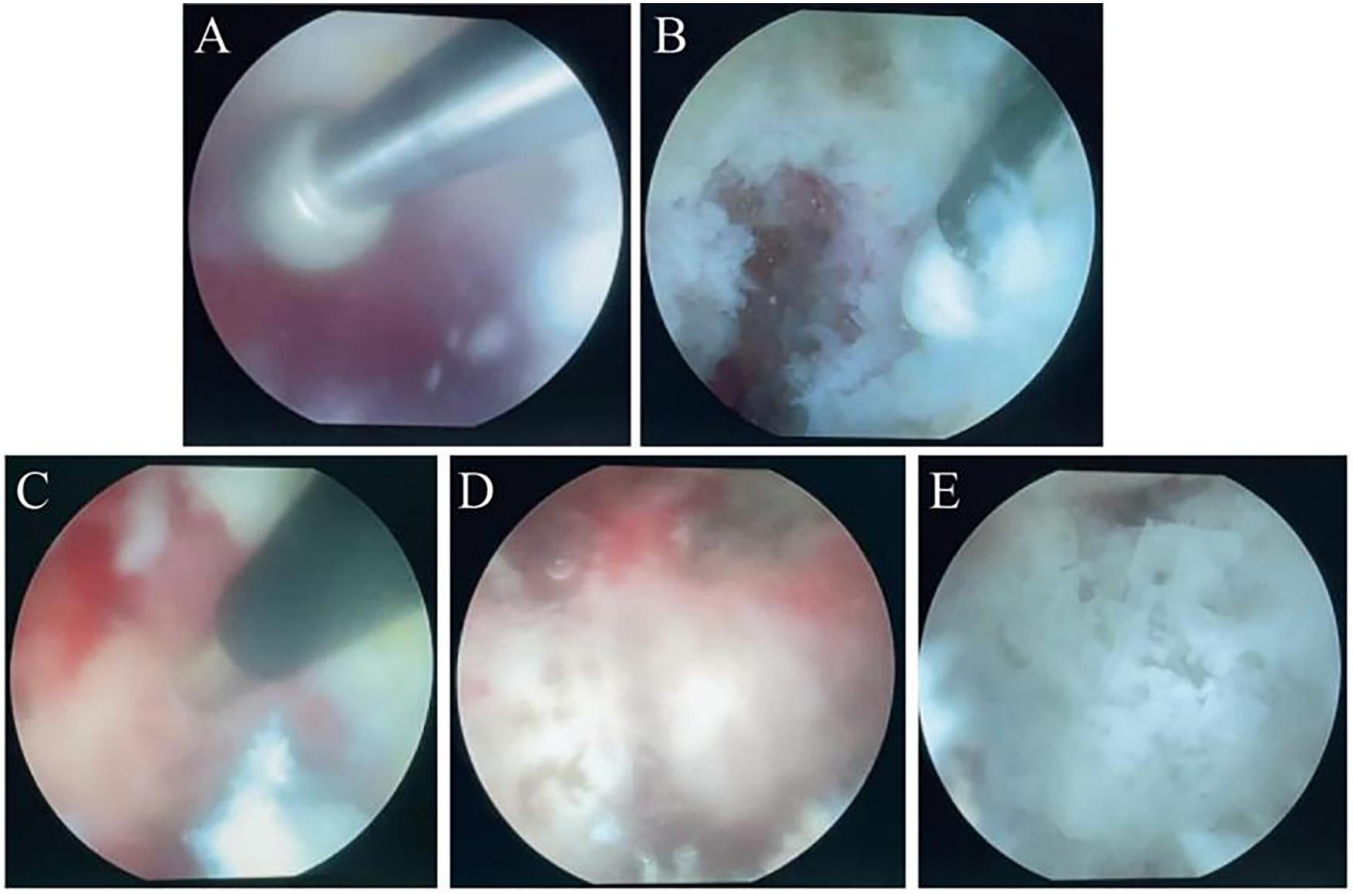
**(A)** Employing a high-speed burr to remove bone. **(B)** Sponge-like tumor tissue is observable. **(C,D)** Excising the tumor lesion until reaching normal bone. **(E)** Implanting allogeneic bone within the lesion.

**Figure 4:**
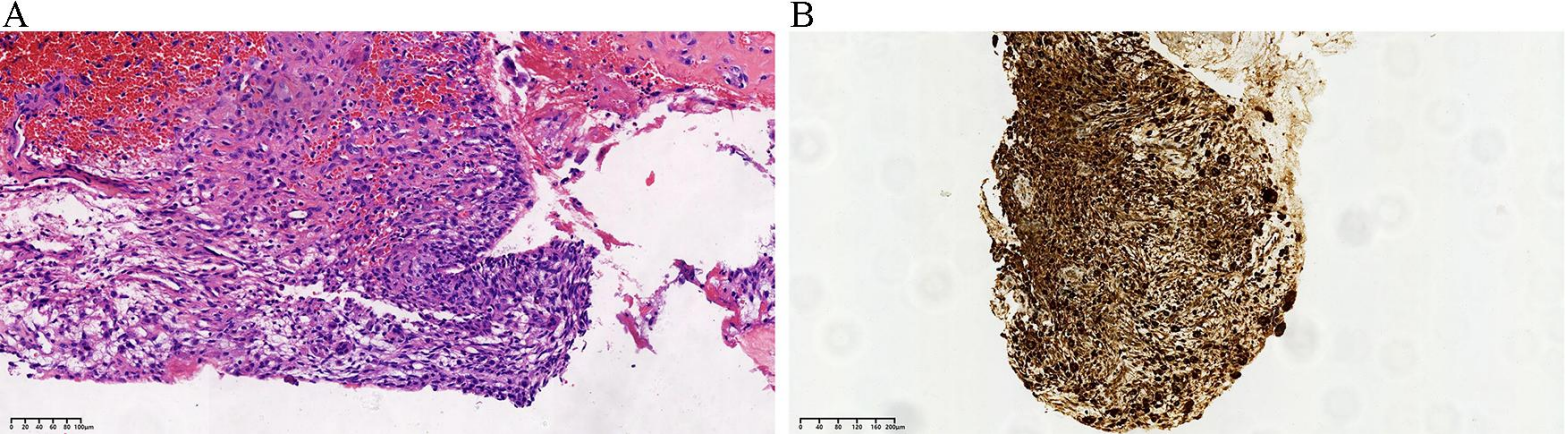
Postoperative pathology suggests benign fibrous histiocytoma. **(A)** Postoperative pathological tissue, stained with HE, reveals fragmented trabecular bone, proliferation of fibroblasts and tissue cells, with focal presence of multinucleated giant cells; **(B)** immunohistochemical staining indicates positive CD68 expression.

**Figure 5:**
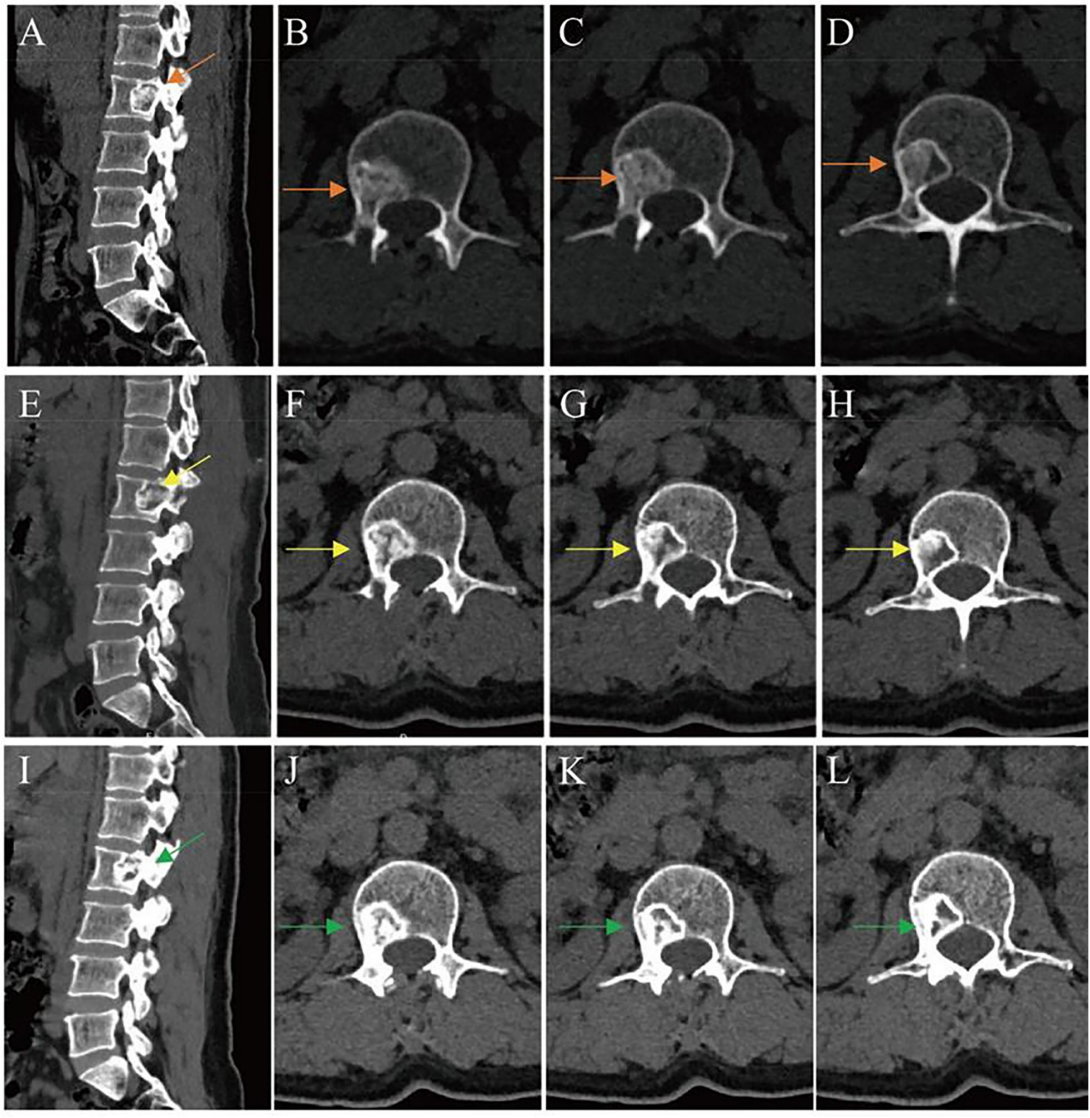
Postoperative CT images. **(A–D)** CT images on the first day after surgery, the lesion area of the L2 vertebra is filled with artificial bone (red arrows). **(E–H)** CT images three months postoperatively, partial bone absorption is observed in the graft area of the L2 vertebra, but no apparent tumor recurrence (yellow arrows). **(I–L)** CT images 2 year postoperatively, the graft area of the L2 vertebra shows bone sclerosis, with no evident tumor recurrence (green arrows).

The patient successfully underwent tumor resection under unilateral biportal endoscopy (UBE) (Figures 2, 3). Histopathological examination confirmed the diagnosis of benign fibrous histiocytoma (Figure 4). Postoperative imaging confirmed complete tumor excision with adequate bone grafting (Figures 5A–D). On postoperative day 1, the patient's VAS score for low back pain improved to 1, and she was able to ambulate independently. She was discharged on postoperative day 3 without adjuvant antitumor therapy.

Computed tomography scans obtained at 3 months and 2 years postoperatively showed no evidence of tumor recurrence in the lesion area. The patient reported no low back pain during follow-up (Figures 5E–L).

## 4 Discussion

Spinal benign tumors are rare, and patients are often asymptomatic or exhibit only mild symptoms. The true occurrence rate of these tumors remains unclear (10). Studies have shown that benign spinal tumors more frequently involve the posterior elements of the vertebral bodies (11). In this study, the histopathological types included benign fibrous histiocytoma, osteoblastoma, osteoid osteoma, aneurysmal bone cyst, simple bone cyst, and fibrous dysplasia, all classified as benign lesions according to the WHO Classification of Tumours of Bone, 5th Edition (12). However, most of these benign tumors exhibit aggressive biological behavior. Intralesional curettage is the standard treatment for symptomatic non-aggressive spinal benign tumors. For patients with spinal instability or neurological deficits, surgical resection of the lesions is required. Total *en bloc* spondylectomy has also been reported for the treatment of spinal benign tumors, demonstrating favorable therapeutic outcomes. However, traditional open surgeries, whether curettage or total spondylectomy, are associated with significant trauma, substantial blood loss, and prolonged postoperative recovery.

The management of spinal benign tumors should be tailored based on tumor staging, anatomical location, and the patient's functional goals. In recent years, minimally invasive surgical approaches have emerged as a focus of clinical research, with the core principle being tumor control while minimizing surgical trauma. Currently, well-established minimally invasive techniques include: Percutaneous thermal ablation (e.g., radiofrequency ablation), primarily used for small lesions such as osteoid osteomas (13). Under CT guidance, precise localization of the nidus is achieved, and thermal energy is applied to induce tumor cell necrosis (14). However, this technique carries a risk of thermal injury to adjacent neural structures. Selective arterial embolization, which may be attempted as a definitive treatment for aneurysmal bone cysts in the absence of neurological deficits or significant spinal instability (15). Studies have demonstrated favorable clinical outcomes (16), though multiple treatment sessions are often required. Image-guided percutaneous sclerotherapy with doxycycline injection, as reported in cases of cervical aneurysmal bone cysts. Among 14 patients treated with this approach, 12 achieved successful cure, yielding an efficacy rate of 85.7% (17). Percutaneous vertebroplasty, which provides rapid pain relief in patients with vertebral hemangiomas, facilitates early mobilization, and improves load-bearing capacity of the vertebral body. It is an optimal option for patients without neural compression, though caution is warranted against cement leakage (18). These minimally invasive techniques play a crucial role in symptom control and functional preservation for non-aggressive spinal benign tumors. However, their primary limitation lies in the inability to achieve complete tumor eradication. For benign spinal tumors requiring resection to minimize recurrence risk-particularly those classified as stage S3 in the Enneking Surgical Staging System-conventional minimally invasive approaches are often inadequate, necessitating more invasive open surgeries.

Percutaneous spinal endoscopic surgery, a minimally invasive technique primarily categorized into uniportal endoscopy (UE) and unilateral biportal endoscopy (UBE), has been widely adopted in clinical practice. It is predominantly employed for the treatment of spinal degenerative diseases, including lumbar disc herniation and lumbar spinal stenosis, with its core principle being minimally invasive decompression (19). With advancements in endoscopic technology and instrumentation, its indications continue to expand. Telfeian et al. reported the successful application of UE in four patients with metastatic spinal tumors presenting severe neural compression, demonstrating satisfactory postoperative outcomes with complete resolution of neurological symptoms (20). Shibuya documented the use of UE for lumbar aneurysmal bone cysts (ABCs), with significant alleviation of low back pain postoperatively. Follow-up CT and MRI at 3 years revealed no local recurrence, supporting UE as an effective therapeutic option for ABCs due to its minimal invasiveness, clear visualization, and rapid recovery (21). Our team recently conducted a retrospective study involving 15 patients with benign spinal tumors treated with UE, all of whom exhibited marked clinical improvement postoperatively without tumor recurrence during follow-up, further validating the feasibility and efficacy of spinal endoscopy for benign spinal tumors.

Compared to UE, UBE offers distinct advantages in managing spinal degenerative diseases owing to its dual-channel independent operation and broader instrument maneuverability (22). Although sporadic case reports have highlighted UBE's potential in spinal tumor resection (23, 24), its application for benign spinal tumors remains undocumented. Herein, we present the first series of benign spinal bone tumors treated with UBE, achieving favorable clinical outcomes in all cases. The mean operative time was 77.5 ± 21.2 min, with an average intraoperative blood loss of 17.5 ± 11.7 mL. Pre-discharge CT confirmed adequate lesions clearance, and all patients were followed up for 10–30 months (mean: 16.6 ± 5.4 months). Significant improvements in back pain visual analog scale (VAS) scores were observed at postoperative day 1, 3 months, 6 months, and final follow-up compared to preoperative baselines. Our preliminary data suggest that UBE is a safe and effective surgical approach for benign spinal tumors.

In our view, unilateral biportal endoscopic spinal surgery (UBESS) represents an established minimally invasive spinal endoscopic technique. Unlike single-portal spinal endoscopy, this approach features both an observation channel and an operational channel. The operational channel accommodates conventional open surgical instruments, enabling more flexible and efficient resection of spinal lesions. The application of UBE (unilateral biportal endoscopy) technology for treating non-aggressive spinal benign tumors demonstrates unique advantages. Benign bone tumors maintain stable pathological morphology over extended periods, with rare occurrences of metastasis or malignant transformation. Local excision typically achieves satisfactory therapeutic outcomes (25), thus rendering minimally invasive local resection of benign bone tumors clinically feasible. First, unlike conventional open surgery, UBE technology does not require extensive paraspinal muscle dissection. This minimizes soft tissue trauma, reduces postoperative myogenic low back pain, and facilitates faster patient recovery (26). Second, the entire surgical procedure is performed intralesionally without breaching the cortical bone, thereby preserving spinal stability and eliminating the need for supplemental internal fixation for spinal reconstruction. UBE procedures are conducted in a fluid medium, and the magnified endoscopic visualization provides clear and expansive surgical fields. This significantly enhances the surgeon's capacity for lesions observation and management.

The authors propose the following recommendations for utilizing Unilateral Biportal Endoscopy (UBE) in the management of non-aggressive spinal benign tumors. First, the application of UBE for spinal tumor resection differs from that for degenerative spinal conditions. If the surgeon is relatively unfamiliar with the endoscopic anatomy involved, disorientation may occur during the procedure, necessitating repeated fluoroscopy for localization. To address this, we recommend the use of an “anchor technique” (27, 28), in which Kirschner wires are employed to mark the surgical target and trajectory, thereby guiding the operative process. Second, as the lesions are neoplastic, intraoperative bleeding is common, leading to impaired endoscopic visibility (“blood blindness”), which may increase surgical risk and prolong operative time. Conventional radiofrequency ablation often proves insufficient for hemostasis. Studies have shown that hydrogen peroxide can reduce intraoperative bleeding and prevent postoperative infection (29). In our practice, hydrogen peroxide and absolute alcohol are applied to the lesions to achieve hemostasis, infection prevention, and tumor inactivation. However, the most effective hemostatic measure remains complete lesions removal. Meanwhile, hydrogen peroxide has been demonstrated to induce apoptosis and reduce the recurrence rate (30). Additionally, preventing local tumor recurrence is a critical concern. Radical tumor resection is the primary measure to minimize recurrence. We recommend using a high-speed burr to extensively excise the lesions until normal bone is reached, thereby achieving extended curettage. Research indicates that elevating local temperature to 41 °C–45 °C is sufficient to kill bone tumor cells (31). The radiofrequency electrode used in our procedure generates localized temperatures of 40–70 °C, effectively inactivating residual tumor cells within the cavity. Alcohol also exerts tumoricidal effects, aiding in tumor control (32). Ulici et al. reported that 95% alcohol is an effective treatment for aneurysmal bone cysts (33). Following thorough lesions resection, we apply absolute alcohol for chemical inactivation of residual tumor cells to further reduce recurrence. After tumor removal, we recommend filling the osseous defect with allogeneic bone grafts. Finally, due to the constraints imposed by the bony anatomy in endoscopic spinal tumor surgery, excessively large or deep lesions may hinder operative feasibility. The UBE technique has specific requirements regarding the extent of spinal involvement. Our recommendations are as follows: (1) The lesions should primarily involve the posterior spinal elements, such as the pedicle or transverse process. (2) For lesions involving the vertebral body, the tumor should be confined to one side, with the affected area limited to the middle-posterior portion of the vertebral body.

The use of unilateral biportal endoscopy (UBE) for non-aggressive spinal benign tumors presents certain limitations. Unlike discectomy, which often only requires the removal of compressive structures to alleviate clinical symptoms, tumor resection necessitates thorough and complete excision to minimize the risk of postoperative recurrence. The endoscopic approach differs from open surgery in terms of macroscopic visualization, which may lead to a “one-sided view” phenomenon, potentially obscuring critical areas. However, with accumulated surgical experience and technical refinement, skilled surgeons can mitigate this issue. Another limitation involves bone grafting after tumor curettage. The efficiency of endoscopic bone grafting is relatively low, which may result in insufficient graft placement and compromise long-term bony fusion.

In this study, UBE demonstrated satisfactory short-term clinical outcomes in the treatment of non-aggressive spinal benign tumors, characterized by minimal invasiveness, rapid recovery, and reliable efficacy. However, due to the low occurrence rate of spinal benign tumors, the limited sample size from a single center, short follow-up duration, and lack of comparative studies, further multicenter investigations with extended follow-up periods are required to evaluate long-term tumor recurrence. Nonetheless, the authors believe that UBE represents a significant advancement in minimally invasive orthopedic principles and serves as a valuable exploration in the minimally invasive surgical management of non-aggressive spinal benign tumors.

## 5 Conclusions

UBE demonstrates favorable clinical efficacy in the treatment of non-aggressive spinal benign tumors, achieving satisfactory lesions clearance. It is a safe and effective surgical option.

## Data Availability

The raw data supporting the conclusions of this article will be made available by the authors, without undue reservation.
